# Diet quality as a predictor of cardiometabolic disease–free life expectancy: the Whitehall II cohort study

**DOI:** 10.1093/ajcn/nqz329

**Published:** 2020-01-11

**Authors:** Hanna Lagström, Sari Stenholm, Tasnime Akbaraly, Jaana Pentti, Jussi Vahtera, Mika Kivimäki, Jenny Head

**Affiliations:** 1 Department of Public Health, University of Turku and Turku University Hospital, Turku, Finland; 2 Centre for Population Health Research, University of Turku, Turku, Finland; 3 Inserm, U1198, Université Montpellier, École Pratique des Hautes Études, Montpellier, France; 4 Department of Psychiatry and Autism Resources Centre, University Research and Hospital Center of Montpellier, Montpellier, France; 5 Department of Epidemiology and Public Health, University College London, London, United Kingdom; 6 Clinicum, Faculty of Medicine, and Helsinki Institute of Life Science, University of Helsinki, Helsinki, Finland

**Keywords:** Alternative Healthy Eating Index, disease-free expectancy, life expectancy, dietary habits, cardiometabolic disease–free

## Abstract

**Background:**

Poor diet quality has been linked to increased risk of many chronic diseases and premature mortality. Less research has considered dietary habits in relation to disease-free life expectancy.

**Objectives:**

Our objective was to investigate the association of diet quality with cardiometabolic disease–free life expectancy between ages 50 and 85 y.

**Methods:**

Diet quality of 8041 participants of the Whitehall II cohort study was assessed with the Alternative Healthy Eating Index 2010 (AHEI-2010) in 1991–1994, 1997–1999, and 2002–2004. The measurement of diet quality closest to age 50 for each participant was used. We utilized repeat measures of cardiometabolic disease (coronary heart disease, stroke, and type 2 diabetes) from the first observation when participants were aged ≥50 y. Multistate life table models with covariates age, gender, occupational position, smoking, physical activity, and alcohol consumption were used to estimate total and sex-specific cardiometabolic disease–free life expectancy from age 50 to 85 y for each AHEI-2010 quintile, where the lowest quintile represents unhealthiest dietary habits and the highest quintile the healthiest habits.

**Results:**

The number of cardiometabolic disease–free life-years after age 50 was 23.9 y (95% CI: 23.0, 24.9 y) for participants with the healthiest diet, that is, a higher score on the AHEI-2010, and 21.4 y (95% CI: 20.6, 22.3 y) for participants with the unhealthiest diet. The association between diet quality and cardiometabolic disease–free life expectancy followed a dose–response pattern and was observed in subgroups of participants of different occupational position, BMI, physical activity level, and smoking habit, as well as when participants without cardiometabolic disease at baseline were excluded from analyses.

**Conclusions:**

Healthier dietary habits are associated with cardiometabolic disease–free life expectancy between ages 50 and 85.

## Introduction

Poor diet is among the leading modifiable risk factors of premature mortality (1, 2). Single dietary components such as whole grains, fruits and vegetables, nuts, and fish have been found to be associated with a lower risk, whereas high-fat products and red and processed meats are associated with increased risk of all-cause mortality and several noncommunicable diseases (3–6), although not unanimously (7). In addition, measures of healthy dietary patterns assessed through a-priori diet indices or factor analysis–derived patterns have been associated with lower risk of type 2 diabetes (8, 9), coronary heart disease and stroke (10), premature death from cardiovascular disease (CVD) (8, 11, 12), and cancer mortality (13, 14).

Health expectancy has been proposed as a useful summary measure of a population's health that expresses the average number of years that a person can expect to live in “full health” by taking into account years lived in less than full health due to disease and/or disability. Because health expectancy captures both the “quantity” and “quality” of lived years by considering simultaneously both health and years of life lost, it is more informative than life expectancy (LE) alone and allows comparisons of the proportion of life spent in good health or without diseases across different population groups (15).

Characteristics of a healthy lifestyle, such as physical activity, nonsmoking, normal weight, and good sleep, have been shown to contribute to healthy and disease-free LE (16–21). However, less research has considered the role of overall diet using diet quality indices in relation to health expectancy, and very few studies have focused on disease-free LE owing to specific disease groups. Dietary factors are associated especially with CVDs (22, 23) and type 2 diabetes (9). Therefore, it is of interest to examine the extent to which dietary habits are associated with cardiometabolic disease–free LE and whether these associations vary by gender (24, 25).

In the present study, we examined the association of a diet quality index, the Alternative Healthy Eating Index 2010 (AHEI-2010), with cardiometabolic disease–free LE by gender and socioeconomic position in the UK Whitehall II (WHII) study cohort. This outcome was defined as the estimated number of years lived free of coronary heart disease, stroke, and type 2 diabetes.

## Methods

### Study population

This study used data from the WHII study, an ongoing prospective cohort of 10,308 (6895 men and 3413 women) UK civil servants aged 35 to 55 y at study induction (phase 1: 1985–1988) (26). Since phase 1, subsequent phases of data collection have taken place approximately every 2–3 y and alternated between postal questionnaire alone [phase 2 (1989–1990), phase 4 (1995–1996), phase 6 (2001), phase 8 (2006), and phase 10 (2011)] and postal questionnaire accompanied by a clinical examination follow-up phases (phase 3 (1991–1993), phase 5 (1997–1999), phase 7 (2003–2004), phase 9 (2007–2009), and phase 11 (2012–2013)] with response rates ranging from 61% to 79%. In the current study, dietary assessment data were available at phases 3, 5, and 7, and we took the measure of diet closest to age 50 y for each participant. We analyzed repeat measures of cardiometabolic disease from the first observation with valid data when participants were aged ≥50 y (*n* = 8041). A flow chart summarizing inclusion and exclusion criteria for the present study samples is presented in **Supplemental Figure 1**. The University College London Ethics Committee approved the study, and written informed consent was obtained from all participants.

### Dietary assessment

A machine-readable FFQ based on one used in the US Nurses’ Health Study was sent to the participants (27).The food list (127 items) in the FFQ was anglicized, and foods commonly eaten in the United Kingdom were added. A common unit or portion size for each food was specified, and participants were asked how often, on average, they had consumed that amount of the item during the previous year. Response to all items was on a 9-point scale, ranging from “never or less than once per month” to “six or more times per day.” The selected frequency category for each food item was converted to a daily intake. Details of analysis to estimate nutrients and total energy intake are provided elsewhere (28). The validity and reliability of this FFQ in terms of nutrient and food consumption have been documented in detail elsewhere (29).

Participants with an incomplete FFQ (>10% missing items) and participants with unreasonably high (>3500 kcal/d for women and >4200 kcal/d for men) or low (<500 kcal/d for women and <800 kcal/d for men) intakes were excluded to avoid influence of outliers. The FFQ data were used to assess diet quality using the AHEI-2010 (8). The AHEI-2010 is based on 11 components: 6 components for which the highest intakes were supposed to be ideal [vegetables, fruit, whole grains, nuts and legumes, long chain n–3 fats, and PUFAs (excluding long-chain ω-3 PUFAs)], 1 component for which only moderate intake was supposed to be ideal (alcohol), and 4 components for which avoidance or lowest intake were supposed to be ideal (sugar-sweetened drinks and fruit juice, red and processed meat, *trans*-fats, and sodium). Each component is given a minimal score of 0 and a maximal score of 10, with intermediate values scored proportionally, and has the potential to contribute 0–10 points to the total score. All the component scores are summed to obtain a total AHEI-2010 score, which ranges from 0 to 110, with a higher score representing a healthier diet.

In the analyses the AHEI-2010 total score was categorized into quintiles, where the lowest quintile (Q1) represented the unhealthiest dietary habits and the highest quintile (Q5) represented the healthiest habits. The AHEI-2010 cut points in the lowest and in the highest quintile scores were 44 and 61 (range 0–110), respectively. A modified AHEI-2010 score without alcohol intake component was also computed (range 0–100) to assess the extent to which association of diet quality with cardiometabolic disease–free LE is independent of drinking habits.

### Outcome measures

Our LE analyses were conditional on reaching the age of 50 and truncated at age 85; thus, instead of estimating total LE, we estimated partial LE between ages 50 and 85. Partial LE was further divided into healthy and unhealthy LE, and we defined the health expectancy outcome as cardiometabolic disease–free (without coronary heart disease, stroke, and type 2 diabetes) LE based on the occurrence of these diseases between ages 50 and 85. In addition, we took into account mortality when modeling health expectancies. Mortality was ascertained from linked register data with follow-up censored on December 31, 2013 (the year that phase 11 data collection ended).

Cardiometabolic disease–free LE was defined based on the years without these chronic diseases. Incidence of cardiometabolic disease (≥1 of coronary heart disease, stroke, and type 2 diabetes) was ascertained at each observation point from self-reports of doctor-diagnosed disease, clinical screening, and/or medical records. Coronary heart diseases included diagnosed nonfatal myocardial infarction and “definite” angina. Nonfatal myocardial infarction was defined following Multinational Monitoring of Trends and Determinants in Cardiovascular Disease criteria (30) based on study electrocardiograms, hospital acute electrocardiograms, and cardiac enzymes and validated using discharge diagnoses from National Health Service Hospital Episode Statistics data or General Practitioner confirmation up to the end of phase 11. Our measure of coronary heart diseases only included self-reports of nonfatal myocardial infarction or angina if they were subsequently validated in medical records. Stroke was determined from self-report of doctor-diagnosed stroke that was validated in general practice or hospital medical records up to the end of phase 9. Incidence of stroke after phase 9 to end of phase 11 was ascertained from hospital inpatient discharge diagnosis (International Classification of Diseases 10 codes I60, I61, I63, and I64) obtained from linkage with Hospital Episode Statistics inpatient data (31). Diagnosis of type 2 diabetes was defined as either self-reported doctor-diagnosed diabetes, use of antidiabetic medication, or fasting glucose concentration ≥7.0 mmol/L (126 mg/dL). Prevalence of cardiometabolic disease at the first observation point in our study included disease diagnosed at age <50 y from available information on respondents.

### Sociodemographic, BMI, and health behavior characteristics

Sociodemographic variables consisted of age, gender, and occupational position with the use of current (or last, for retired participants) UK civil service employment grade, defined on the basis of salary and grouped into 3 categories: high (senior administrators), middle (executives, professionals, and technical staff), and low (clerical and office support staff) grades. BMI (kg/m^2^) was categorized as normal weight (<25) or overweight (≥25). Health behaviors included physical activity [classified as “active” (≥2.5 h/wk of moderate physical activity or ≥1 h/wk of vigorous physical activity) or “inactive” (<2.5 h/wk of moderate physical activity or <1 h/wk of vigorous physical activity)], smoking (classified as “nonsmoker” or “current smoker”), and alcohol consumption (classified as “nondrinkers,” “1–14 units/wk,” and “>14 units/wk”).

### Statistical methods

Descriptive statistics for our analysis sample are presented based on the time of diet measurement for participants (phase 3, 5, or 7 diet measure closest to age 50). We applied discrete-time multistate life table models to longitudinal data (32). For cardiometabolic disease–free LE, 3 health states were defined (no disease, disease, and dead) and there were 3 possible transitions (no disease to disease, no disease to death, and disease to death).

In the first step of the multistate life table analyses, multinomial logistic regression models were fitted to estimate age-specific transition probabilities of moving between disease states according to the AHEI-2010 score. Each individual contributed data from their age at first measurement of cardiometabolic status. These multinomial logistic models included age in years, gender, occupational position, smoking, physical activity, and alcohol consumption as covariates. Parameter estimates from these models were used to calculate age-specific (single years) transition probabilities between disease states by diet quality and gender. In the second step of analyses, partial LE and chronic disease–free LE from ages 50 to 85 (in total 36 y) were calculated based on these estimated transition probabilities using a stochastic (microsimulation) approach (32). Individual trajectories of cardiometabolic disease from ages 50 to 85 for a simulated cohort of 100,000 persons were generated with distributions of covariates at the starting point based on the observed study-specific prevalence by 5-y age group and gender. Partial LE, and cardiometabolic disease–free LE from ages 50 to 85 were then calculated as the average from these trajectories for AHEI-2010 and gender. Computation of 95% CIs (from 2.5th to 97.5th percentiles) for these multistate life table estimates was performed using a bootstrap method with 500 replicates for the whole analysis process (multinomial analysis and simulation steps). To further examine the potential confounding role of BMI and other lifestyle habits on the association between diet and cardiometabolic disease–free LE, we conducted additional analyses estimating proportion of years spent without cardiometabolic diseases by BMI status, physical activity level, smoking, and alcohol consumption habits. In addition, to assess the role of baseline cardiometabolic disease on the cardiometabolic disease–free LE estimates, supplementary analyses were conducted after excluding participants with cardiometabolic disease at baseline. To examine the extent to which AHEI-2010–related transitions to poor health and death differ by gender and socioeconomic position, we repeated analyses including interactions between AHEI-2010, gender, and socioeconomic position in multinomial logistic models. Furthermore, to examine the role of alcohol on the cardiometabolic disease–free LE estimates, we conducted additional analyses by the modified AHEI-2010 dietary index and by stratifying the results by 3 alcohol intake groups (“nondrinkers,” “1–14 units/wk,” and “>14 units/wk”). Finally, the proportion of life spent without cardiometabolic diseases between ages 50 and 85 was calculated by dividing the cardiometabolic disease–free LE with partial LE. All analyses were conducted in SAS 9.2 (SAS Institute Inc.) using the SPACE (Stochastic Population Analysis of Complex Events) program (32).

## Results

Characteristics according to quintile of AHEI-2010 at the time of the diet measurement closest to age 50 y are shown in **Table 1**. Prevalence of cardiometabolic disease at baseline was <10% across AHEI-2010 quintiles. In the lowest AHEI-2010 quintile were more overweight, physically inactive, and current smokers compared with other AHEI-2010 quintiles among both men and women. Fewer men (18%) than women (21%) were in the highest quintile of AHEI-2010 (reflecting a healthier diet).

**TABLE 1:** Characteristics of participating men and women by Alternative Healthy Eating Index 2010 quintiles (total range from 0 to 110) at the time of first observation closest to age 50 y (from phases 3 or 5 or 7)

	Alternative Healthy Eating Index 2010 scores				
Characteristic	Q1 (22 to 44; unhealthiest)	Q2 (44.5 to 49.5)	Q3 (50.0 to 55.0)	Q4 (55.5 to 61.0)	Q5 (61.5 to 91.0; healthiest)
Men (*n* = 5543)					
Sample size, % (*n*)	22 (1224)	19 (1068)	23 (1225)	19 (1030)	18 (996)
Mean age ± SD, y	53.0 ± 3.2	53.2 ± 3.3	53.3 ± 3.3	53.5 ± 3.4	53.4 ± 3.4
Cardiometabolic disease, %	8.7	9.6	9.0	9.0	9.4
Occupational position, %					
High	34.7	38.3	43.7	42.8	42.6
Middle	55.4	54.7	50.0	51.1	50.8
Low	9.9	7.0	6.4	6.1	6.6
Overweight (BMI >25 kg/m^2^), %	54.6	50.2	49.3	50.9	40.3
Physically inactive,^1^ %	48.9	41.9	39.8	38.5	37.8
Current smokers, %	20.7	12.5	11.2	7.8	6.2
Alcohol consumption, %					
Nondrinkers	17.7	17.6	12.2	10.2	11.4
Moderate (1–14 units/wk)	37.5	47.9	56.3	62.9	65.7
Heavy (>14 units/wk)	44.9	34.5	31.6	26.9	23.0
Women (*n* = 2498)					
Sample size, % (*n*)	19 (473)	19 (465)	20 (500)	22 (543)	21 (517)
Mean age ± SD, y	54.1 ± 3.6	53.8 ± 6.6	53.9 ± 3.5	53.8 ± 3.6	53.4 ± 3.4
Cardiometabolic disease, %	9.3	8.6	7.2	7.9	7.5
Occupational position, %					
High	8.7	8.8	15.2	13.1	16.8
Middle	37.0	41.7	42.0	43.5	46.8
Low	54.3	49.5	42.8	43.5	36.4
Overweight (BMI >25), %	60.7	51.2	51.2	45.1	41.8
Physically inactive,^1^ %	70.8	64.1	64.2	60.0	60.2
Current smokers, %	26.4	17.6	16.2	14.0	7.5
Alcohol consumption, %					
Nondrinkers	39.8	32.0	28.8	25.1	26.1
Moderate (1–14 units/wk)	46.7	57.0	59.4	68.0	69.1
Heavy (>14 units/wk)	13.5	11.0	11.8	7.0	4.8

1 Physical inactivity (vigorous physical activity <1 h/wk and/or moderate physical activity <2.5 h/wk).

Results for the estimated partial LE and cardiometabolic disease–free LE between ages 50 and 85 by AHEI-2010, total study population, and separately by gender are shown in **Table 2**. A graded relation of more years without cardiometabolic diseases with higher AHEI-2010 levels was observed in general as well as for both men and women. In the total study population, cardiometabolic disease–free LE was 2.5 y longer in the highest AHEI-2010 compared with the lowest AHEI-2010 group, the absolute years being 32 for highest and 30 for the lowest quintile. Men with the highest AHEI-2010 lived 2.2 y longer and women 2.8 y longer without cardiometabolic diseases compared with the lowest AHEI-2010 groups (*P* value for gender interaction >0.05).

**TABLE 2:** Partial life expectancy and cardiometabolic disease–free life expectancy between ages 50 and 85 y by Alternative Healthy Eating Index 2010 (AHEI-2010)

		Partial life expectancy^1^		Cardiometabolic disease–free life expectancy	
	AHEI-2010	Years	95% CI	Years	95% CI
Total (*n* = 8041)	Q1 (unhealthiest)	30.17	29.60, 30.79	21.44	20.59, 22.29
	Q2	31.28	30.70, 31.91	22.78	22.09, 23.63
	Q3	32.02	31.40, 32.49	23.81	22.94, 24.43
	Q4	31.80	31.40, 32.49	24.17	23.26, 25.01
	Q5 (healthiest)	32.07	31.40, 32.69	23.94	23.04, 24.85
Men (*n* = 5543)	Q1 (unhealthiest)	30.15	29.50, 30.80	21.17	20.25, 21.96
	Q2	31.15	30.50, 31.91	22.34	21.60, 23.33
	Q3	31.97	31.30, 32.51	23.40	22.53, 24.17
	Q4	31.71	31.10, 32.37	23.53	22.71, 24.58
	Q5 (healthiest)	31.95	31.30, 32.61	23.34	22.31, 24.34
Women (*n* = 2498)	Q1 (unhealthiest)	30.23	29.60, 31.17	22.28	21.08, 23.42
	Q2	31.63	30.80, 32.26	23.93	23.09, 25.07
	Q3	32.16	31.40, 32.65	24.93	23.64, 25.07
	Q4	31.97	31.10, 32.71	25.44	24.09, 26.40
	Q5 (healthiest)	32.30	31.60, 33.00	25.08	23.83, 26.07

1 Partial life expectancy is life expectancy between ages 50 and 85 y; estimated from models with covariates age, gender, occupational position, smoking, physical activity, and alcohol consumption.

**FIGURE 1:**
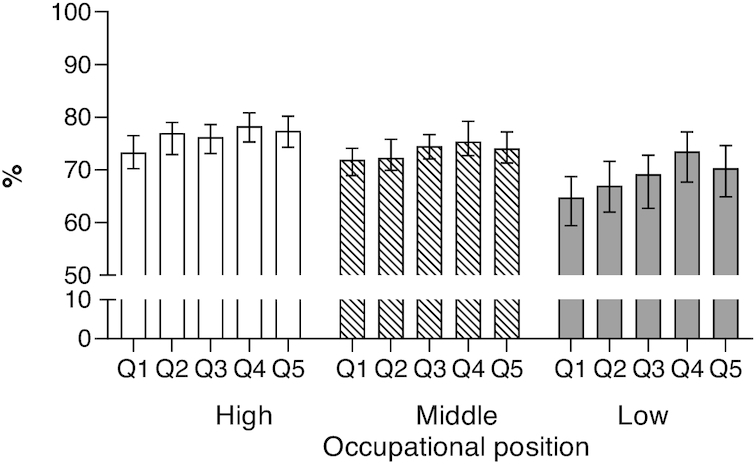
Proportion (95% CI) of life spent without cardiometabolic diseases between ages 50 and 85 y by Alternative Healthy Eating Index 2010 by occupational position. Number of participants = 8041. Adjusted for age, gender, occupational position, smoking, physical activity, and alcohol consumption; *P* values for interactions >0.05. Q1 = unhealthiest quintile; Q5 = healthiest quintile.

**FIGURE 2:**
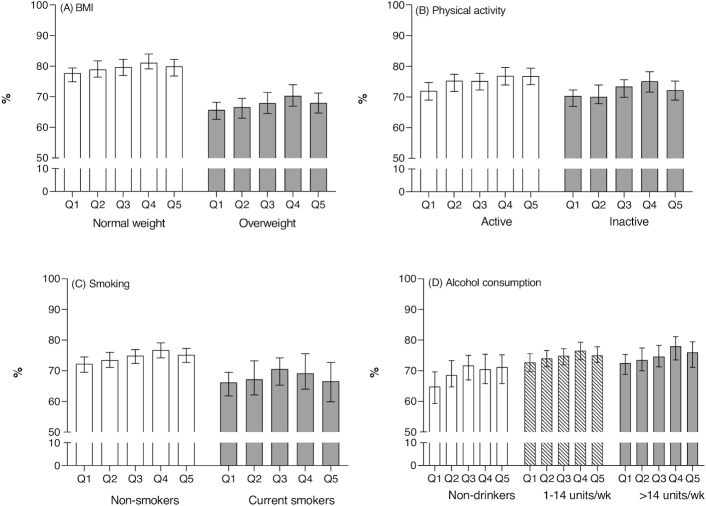
Proportion (95% CI) of life spent without cardiometabolic diseases between ages 50 and 85 y by Alternative Healthy Eating Index 2010 (AHEI-2010) (A) by BMI class, (B) by physical activity, (C) by smoking habits, and (D) by alcohol consumption (modified AHEI-2010, without alcohol component). Number of participants = 8041. Adjusted for age, gender, socioeconomic position, smoking, physical activity, and alcohol consumption; *P* values for all interactions >0.05. Q1 = unhealthiest quintile; Q5 = healthiest quintile.

We also conducted subgroup analyses by occupational position, BMI, physical activity level, smoking, and alcohol consumption. The proportion of years spent without cardiometabolic diseases ranged from 77–78% (2 highest occupational positions and highest AHEI-2010) to 65% (low occupational position and lowest AHEI-2010) (**Figure 1**; *P* values for gender and occupational position interactions >0.05). In addition, the graded relation of diet quality with more years without cardiometabolic diseases was seen among normal weights, physically active, and nonsmokers (**Figure 2**A–C; *P* values for all interactions >0.05). However, the associations were less clear among those who were overweight, physically inactive, and smoking although none of the interactions of diet quality with lifestyle habits were statistically significant. More variation was seen for estimated proportion of years spent without cardiometabolic diseases by drinking habits, although again not statistically significant. The lowest percentage of cardiometabolic disease–free LE was observed in the lowest modified AHEI-2010 (without alcohol component) diet quintile in all drinking habits groups (Figure 2D).

To examine the role of alcohol on the cardiometabolic disease–free LE estimates, we computed a modified AHEI-2010 score without the alcohol component. After including alcohol consumption in the model, results of these analyses showed an attenuation of the association between modified AHEI-2010 score and cardiometabolic disease–free LE in general as well as for both men and women (**Supplemental Table 1**). In general, cardiometabolic disease–free LE was 2.2 y longer in the highest modified AHEI-2010 compared with the lowest modified AHEI-2010. Men with the highest modified AHEI-2010 lived 2.0 y longer and women 1.8 y longer without cardiometabolic diseases compared with the lowest modified AHEI-2010 (Supplemental Table 1).

To examine the role of baseline cardiometabolic disease on the cardiometabolic disease–free LE estimates, we conducted additional analyses by excluding those with cardiometabolic disease at baseline (**Supplemental Table 2**). The results are closely similar to those shown in Table 2. The estimates of cardiometabolic disease–free LE for the total study population, as well as for men and women, are almost 2 y more than for the whole study population (including those having ≥1 cardiometabolic disease at the baseline). Despite this, we still see a graded association between diet and cardiometabolic disease–free LE so that cardiometabolic disease–free LE was 2.5 y longer in the highest AHEI-2010 compared with the lowest AHEI-2010 group, the absolute years being 32 for highest and 30 for the lowest quintile.

To provide more detailed information on the magnitude of risk, the associations between AHEI-2010 and each possible transition are shown in **Table 3**. Unhealthy eating habits were also associated with higher likelihood of moving from disease-free to cardiometabolic disease state, and from disease-free to death so that cardiometabolic disease–free LE was 2.5 y longer in the highest AHEI-2010 compared with the lowest AHEI-2010 group, the absolute years being 32 for highest and 30 for the lowest quintile.

**TABLE 3:** Alternative Healthy Eating Index 2010 quintile-specific ORs for cardiometabolic disease transitions from multinomial logistic models

	Disease-free to cardiometabolic disease^1^		Disease-free to death^1^		Cardiometabolic disease to death^2^	
	OR^3^	95% CI	OR^3^	95% CI	OR^3^	95% CI
Q1 (unhealthiest)	1.15	0.97, 1.36	1.36	1.03, 1.79	1.22	0.83, 1.79
Q2	1.08	0.91, 1.28	1.04	0.78, 1.40	1.30	0.88, 1.91
Q3	0.99	0.84, 1.17	0.98	0.74, 1.30	0.92	0.61, 1.37
Q4	0.90	0.76, 1.07	1.06	0.80, 1.41	0.97	0.64, 1.48
Q5 (healthiest)	1.00		1.00		1.00	

1 OR from multinomial model with “cardiometabolic disease–free” as reference category.

2 OR from multinomial model with “cardiometabolic disease” as reference category.

3 Adjusted for age, gender, occupational position, smoking, physical activity, and alcohol consumption.

## Discussion

In this prospective cohort study of >8000 UK men and women, adherence to healthy diet, assessed by the AHEI-2010, was associated with longer cardiometabolic disease–free LE. Men and women with healthier dietary habits, that is, a higher score on the AHEI-2010, lived ∼2.5 y longer without cardiometabolic diseases than participants with lower scores of AHEI-2010 between ages 50 and 85.

Our results are consistent with previous studies showing that higher diet quality is associated with more years in good health compared with lower diet quality (14, 33–36). However, to our knowledge this is the first study to show the association of adherence to a healthy diet assessed by the AHEI-2010 and longer cardiometabolic disease–free LE, which is an important outcome because diet is known to be an important risk factor for CVDs (37). All in all, the present study extends findings showing that even moderate increases in diet quality are associated with reduced risk of unhealthy aging, which incorporates cardiovascular, metabolic, musculoskeletal, respiratory, mental, and cognitive components (38), lower risk of type 2 diabetes (8, 9, 39), lower prevalence of CVDs (10), and reduced total and CVD mortality (13, 39–44).

We found an association between higher score on the AHEI-2010 and cardiometabolic disease-free LE within different occupational positions. These subgroup differences should be interpreted cautiously because the estimates were imprecise and we did not correct the analyses for multiple testing. However, these differences are plausible because cost is likely to be a significant contributor to healthiness of food choices: people with a lower socioeconomic position might have fewer opportunities to purchase higher-quality foods, which are more expensive (25, 45).

The association between higher score on the AHEI-2010 and cardiometabolic disease–free LE seemed to be independent of BMI and lifestyle factors, including physical activity level and smoking habits. Our results showed additional years of cardiometabolic disease–free LE associated with a higher score on the AHEI-2010 among normal weights, physically active, and nonsmokers. Our results are in line with earlier studies, which have shown that shorter life expectancy without CVD has been linked to obesity (21, 46), physical inactivity (18), and smoking (16). We found mixed results for alcohol consumption in different AHEI-2010 score groups, although a recent study supports limits for alcohol consumption that are much lower than recommended in most current guidelines (47).

A major strength of this study is that it is based on a large prospective cohort study with high response rate and repeated measurements of cardiometabolic diseases over a long follow-up period. The use of microsimulation to estimate cardiometabolic disease–free LE provides internally consistent results. It is a well-known fact that dietary habits are one of the leading modifiable risk factors and their relative importance has increased (48, 49). Analyzing overall diet is useful because it captures potential food and nutrient interactions that studies of single nutritional items cannot, and this allowed us to study in more detail possible relations between diet quality and cardiometabolic disease–free LE in a large cohort of older persons. Previous findings from the WHII study suggesting that high adherence to AHEI or AHEI-2010 was associated with reduced risk of all-cause and cardiovascular mortality (11, 13), long-term inflammation (50), and reduced odds of subsequent recurrent depressive symptoms (12) support the relevance of using AHEI-2010 in the present analysis. It has also been shown that AHEI-2010 captures key aspects of a healthy diet, which allows comparison between studies (51). However, our cohort comprised UK civil servants, potentially reducing the generalizability of our findings. Occupational cohorts are, by their very nature, healthier than the general population, so the range of scores on the AHEI questionnaire and the distribution of cardiometabolic health might be narrower. This being the case, the estimated benefits from a healthy diet reported here will, if anything, be an underestimate of those in the general population, which includes people not in employment.

Potential limitations of our study included the use of self-reported data on dietary behavior, which can lead to a potential misclassification of the exposure. However, self-reported food consumption data are frequently used to study prevalence of differences of dietary practices in large populations, and dietary patterns are moderately stable in adulthood (52). The assessment of dietary intake using a semiquantitative FFQ also constitutes a limitation. This method is less precise than those based on weighted records, but it nevertheless covers a range of specific foods and is feasible for large-scale cohort studies, such as ours. The validity of FFQs has been criticized (53), but they appear to be reasonable in assessing associations of nutrients and food consumption with outcomes, at least in the UK context (27, 29). We have shown, for example, that nutrient intakes estimated by the FFQ method are correlated with biomarker concentrations and intake estimates from the 7-d diary. Although the FFQ is open to measurement errors common to all self-reported dietary assessments (54), it remains a mainstream method of analytical epidemiological studies (53). Indeed, many of the current dietary recommendations and policies to reduce disease burden (e.g., obesity, type 2 diabetes, and CVD) rely on evidence from studies using FFQs (55).

We could not exclude the possibility of reverse causality, namely that low or high adherence to a healthy diet assessed by the AHEI-2010 at the first observational point was a result of chronic/cardiovascular disease(s). However, results for those without cardiometabolic disease at baseline are closely similar to results for the whole study population, suggesting that reverse causality does not explain the findings. Another limitation is the use of only 3 cardiometabolic health conditions, specifically, coronary heart disease, stroke, and type 2 diabetes, to estimate chronic disease–free LE, leaving hypertension and metabolic syndrome out of the analysis. The results of this study are based on microsimulation using estimated transition probabilities and not direct observation of LE.

In conclusion, this study supports a consistent dose–response association between healthy diet quality assessed by the AHEI-2010 and longer healthy and CVD-free LE between ages 50 and 85. Persons with the highest adherence to healthy dietary guidelines lived ∼2.5 y longer without cardiometabolic disease.

## Supplementary Material

nqz329_Online_Supplementary_Material

## Notes

This work was supported by the UK Medical Research Council (MR/K013351/1, G0902037), the British Heart Foundation (RG/13/2/30098), the US National Institutes of Health (R01HL36310, R01AG013196), and the Academy of Finland (286294, 294154 and 319246 to SS; 311492 to MK).

Supplemental Figure 1 and Supplemental Tables 1 and 2 are available from the “Supplementary data” link in the online posting of the article and from the same link in the online table of contents at https://academic.oup.com/ajcn/.

Abbreviations used: AHEI-2010, Alternative Healthy Eating Index 2010; CVD, cardiovascular disease; LE, life expectancy; WHII, Whitehall II study.
