# Supplementary material for: Diet quality as a predictor of cardiometabolic disease–free life expectancy: the Whitehall II cohort study
Source: Am J Clin Nutr. 2020 Jan 11;111(4):787–94. doi: 10.1093/ajcn/nqz329 (PMC7138656; doi:10.1093/ajcn/nqz329)
Supplement: nqz329_Online_Supplementary_Material [file nqz329_online_supplementary_material.docx]

**Diet quality as a predictor of cardiometabolic disease-free life expectancy: the Whitehall II cohort study**

**Lagström H. et al.**

**Online Supplementary Material**

**Supplementary Figure 1.** Flowchart summarizing exclusion and inclusion criteria for present study samples from the Whitehall II cohort study.

| Start Whitehall II 1985-1988:  Phase 1  Baseline present study:  Phase 3 or 5 or 7 | n=10,380  n=9,356  n=8,375  Excluded participants:  Dropped out or died before phase 3, n=1,024  Excluded participants:  Missing, incomplete or energy intake outliers for diet measure at phase 3, 5 and 7, n=981  Excluded participants:  No information on cardiometabolic disease status between ages 50 and 85, n=334  Participants included in analysis with dietary data for first observation closest to age 50 years, n=8,041 |
| --- | --- |

**Supplementary Table 1.** Partial life expectancy and cardiometabolic disease-free life expectancy between ages 50 and 85 years by Alternative Healthy Eating Index 2010 (AHEI 2010) without alcohol component in the AHEI-2010.

|  | **AHEI 2010** | **Partial life expectancy^1^** | | **Cardiometabolic disease-free life expectancy** | |
| --- | --- | --- | --- | --- | --- |
|  |  | Years | 95% CI | Years | 95% CI |
| **Total** | Q1 (unhealthiest) | 30.26 | 29.45, 30.77 | 21.19 | 20.87, 21.89 |
|  | Q2 | 31.60 | 30.92,32.38 | 23.46 | 22.62, 24.84 |
|  | Q3 | 31.77 | 31.37, 32.22 | 23.43 | 22.76, 23.90 |
|  | Q4 | 31.84 | 31.46, 32.18 | 24.25 | 23.58, 24.93 |
|  | Q5 (healthiest) | 31.96 | 31.18, 32.65 | 23.27 | 22.48, 24.31 |
|  |  |  |  |  |  |
| **Men** | Q1 (unhealthiest) | 30.24 | 29.37, 30.70 | 20.79 | 20.29, 21.52 |
|  | Q2 | 31.55 | 30.76, 32.41 | 23.18 | 22.35, 24.48 |
|  | Q3 | 31.69 | 31.21, 32.11 | 22.96 | 22.18, 23.55 |
|  | Q4 | 31.77 | 31.29, 32.22 | 23.77 | 23.10, 24.82 |
|  | Q5 (healthiest) | 31.90 | 30.93, 32.58 | 22.76 | 21.81, 23.65 |
|  |  |  |  |  |  |
| **Women** | Q1 (unhealthiest) | 30.32 | 29.62, 31.09 | 22.55 | 21.70, 24.06 |
|  | Q2 | 31.74 | 30.75, 32.29 | 24.33 | 23.36, 25.68 |
|  | Q3 | 32.01 | 31.50, 32.45 | 24.69 | 24.15, 25.74 |
|  | Q4 | 31.96 | 31.62, 32.59 | 25.15 | 24.22, 26.58 |
|  | Q5 (healthiest) | 32.06 | 31.60, 32.74 | 24.13 | 23.06, 25.35 |

^1^ Partial life expectancy = life expectancy between ages 50 to 85 years; estimated from models with covariates age, gender, occupational position, smoking, physical activity and alcohol consumption.

**Supplementary Table 2.** Partial life expectancy and cardiometabolic disease-free life expectancy without cardiometabolic disease at baseline between ages 50 and 85 years by Alternative Healthy Eating Index 2010 (AHEI-2010).

|  | **AHEI-2010** | **Partial life expectancy^1^** | | **Cardiometabolic disease-free life expectancy** | |
| --- | --- | --- | --- | --- | --- |
|  |  | Years | 95% CI | Years | 95% CI |
| **Total** | Q1 (unhealthiest) | 30.31 | 29.73, 30.88 | 23.35 | 22.57, 24.06 |
|  | Q2 | 31.46 | 30.88, 32.13 | 24.95 | 24.23, 25.66 |
|  | Q3 | 32.08 | 31.44, 32.54 | 25.63 | 24.80, 26.24 |
|  | Q4 | 31.89 | 31.33, 32.50 | 26.02 | 25.38, 26.81 |
|  | Q5 (healthiest) | 32.20 | 31.52, 32.78 | 25.88 | 24.96, 26.66 |
|  |  |  |  |  |  |
| **Men** | Q1 (unhealthiest) | 30.32 | 29.65,30.92 | 23.11 | 22.22,23.83 |
|  | Q2 | 31.35 | 30.73,32.11 | 24.54 | 23.75,25.32 |
|  | Q3 | 32.04 | 31.38,32.55 | 25.26 | 24.41,25.93 |
|  | Q4 | 31.82 | 31.29,32.48 | 25.52 | 25.00,26.42 |
|  | Q5 (healthiest) | 32.07 | 31.38,32.70 | 25.31 | 24.41,26.22 |
|  |  |  |  |  |  |
| **Women** | Q1 (unhealthiest) | 30.29 | 29.71,31.25 | 24.11 | 23.37,25.14 |
|  | Q2 | 31.76 | 30.96,32.43 | 26.03 | 25.05,26.75 |
|  | Q3 | 32.19 | 31.52,32.69 | 26.61 | 25.65,27.36 |
|  | Q4 | 32.02 | 31.15,32.75 | 26.97 | 25.93,27.77 |
|  | Q5 (healthiest) | 32.45 | 31.67,33.07 | 26.96 | 25.89,27.75 |

^1^ Partial life expectancy = life expectancy between ages 50 to 85 years. Results adjusted for age, gender, occupational position, smoking, physical activity and alcohol consumption.
